# Development and characterization of dibenzalacetone-loaded oleogels as a potential photoprotective agents for sunscreen formulations

**DOI:** 10.1038/s41598-026-56234-w

**Published:** 2026-06-09

**Authors:** Nermin M. Sheta, Azza T. Taher, Kareem O. Rashwan, Ashraf M. Mahmoud, Khaled E. Abuelella

**Affiliations:** 1 Department of Pharmaceutics, Faculty of Pharmacy, October 6 University, 6th of October City, Giza, 12585 Egypt; 2 Department of Pharmaceutical Organic Chemistry, Faculty of Pharmacy, Cairo University, Cairo, Egypt; 3 Department of Pharmaceutical Organic Chemistry, Faculty of Pharmacy, October 6 University, 6th of October City, Giza, 12585 Egypt

**Keywords:** Dibenzalacetone, Oleogel, Photoprotection, Sunscreen, Rheological analysis, Cytotoxicity evaluation, Chemistry, Drug discovery, Materials science

## Abstract

Prolonged exposure to ultraviolet radiation induces erythema, accelerates photoaging, and increases the risk of skin cancer. Sunscreens are the primary strategy for preventing UV-induced skin damage; however, the effective topical delivery of photoprotective agents is often limited by formulation challenges. Dibenzalacetone (DBA), a chalcone derivative with broad UVA and UVB absorption, exhibits strong photoprotective potential but is restricted by poor aqueous solubility. This study aimed to develop and optimize DBA-loaded silicon dioxide (SD) oleogels as topical photoprotective systems. Oleogels were prepared using argan oil (AO) or jojoba oil and optimized through a 2³ factorial design. DBA solubility, critical gelation concentration, rheological behavior, drug content, and in vitro sun protection factor (SPF) were evaluated. Further characterization included spreadability, photostability, skin permeation, differential scanning calorimetry, Fourier transform infrared spectroscopy, stability studies, and cytotoxicity assessment on human keratinocytes. DBA demonstrated high solubility in both oils, enabling the formation of stable, translucent oleogels at a structuring agent concentration of 10%. All formulations exhibited non-Newtonian, pseudoplastic, and thixotropic behavior, acceptable drug content, and broad-spectrum UV protection (λc > 370 nm). AO-based oleogels showed superior SPF values. Formulation F8 (AO, 15% SD, 8% DBA) achieved the highest SPF, excellent spreadability, enhanced photostability, low skin permeation, and no cytotoxicity up to 400 µg/mg.

**Supplementary Information:**

The online version contains supplementary material available at 10.1038/s41598-026-56234-w.

## Introduction

The skin is a complex barrier system consisting of surface keratinocytes, inter-keratinocytes, dermis, and subdermal structures. It is the first line of defense to protect our bodies from external disturbances such as UV exposure, chemical and mechanical strain, and infection^1^. Prolonged and repeated exposure of the skin to sunlight can induce both immediate and long term structural alterations^2^. In the short term, ultraviolet radiation (UVR) causes erythema, a condition characterized by skin reddening, commonly known as sunburn. This inflammatory response results from increased blood flow to the affected area due to UV-induced damage to epidermal cells. Erythema is subsequently followed by the activation of melanocytes, which respond by increasing melanin synthesis, leading to skin darkening, commonly referred as tanning^3^. Prolonged and repeated UV exposure, however, has more severe long-term consequences, including irreversible degradation of skin elasticity, which accelerates photoaging. Moreover, chronic UV-induced damage significantly increases the risk of skin cancer, encompassing both melanoma and non-melanoma types^4^. The use of sunscreens as a protective measure against the harmful effects of solar radiation has increased significantly over the past few decades. This observation can be attributed to a growing awareness of the adverse consequences of prolonged sun exposure, including photoaging and skin cancer. Public health initiatives and government led awareness campaigns have also contributed to the widespread approval of sunscreen, emphasizing its role in skin protection and long-term health benefits^5^.

**Fig. 1:**
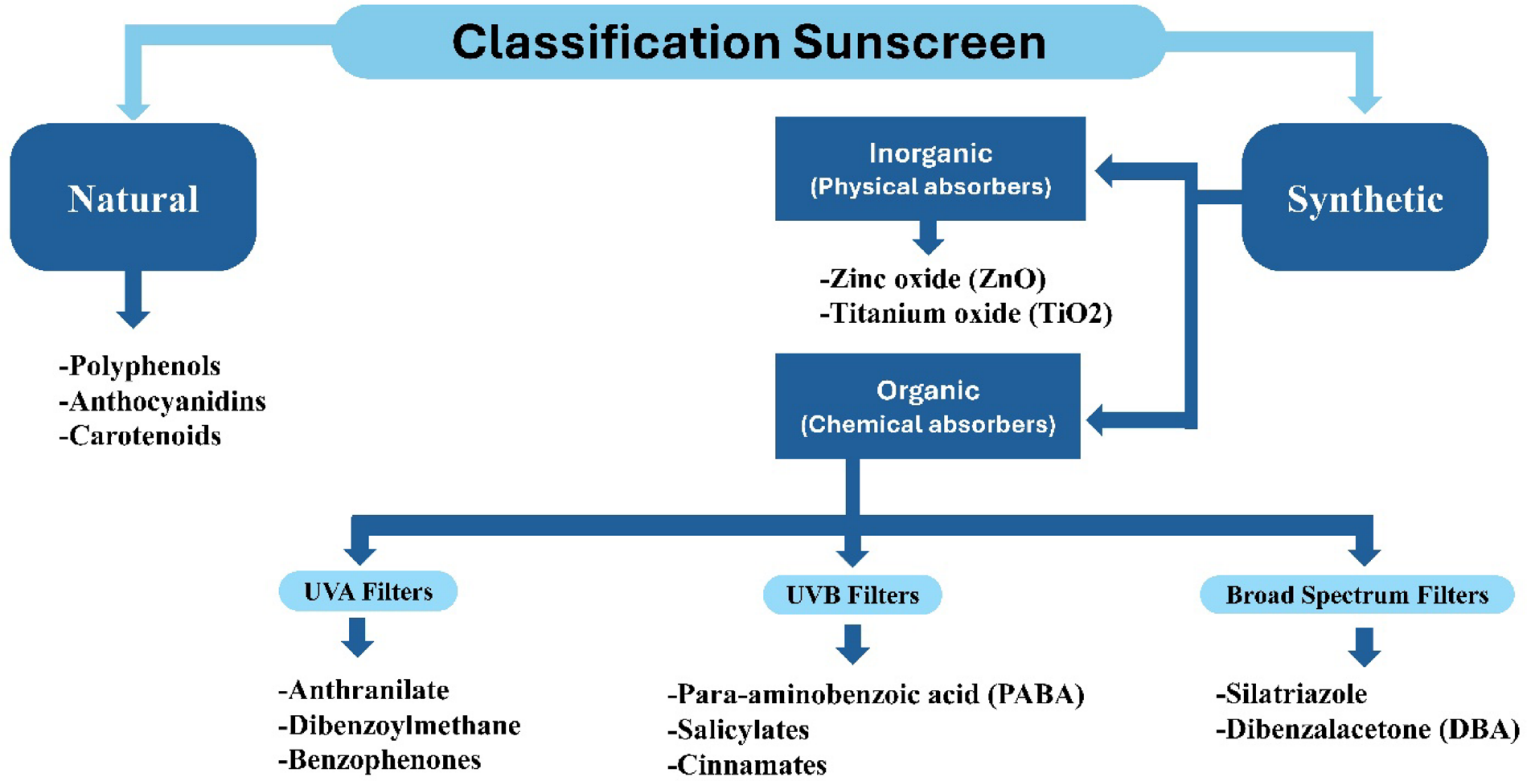
Classification of sunscreen.

Sunscreens are classified into various types, including natural or synthetic (organic and inorganic sunscreens) (Fig. 1)^6^. Natural sunscreens, including polyphenols, anthocyanidins, carotenoids, various vitamins and triglyceride oils derived from fruits, vegetables, lichens and algae have demonstrated superior efficacy, as reported by Resende et al.^7^, Kuthi et al.^8^., and Romes et al.^9^. Their effectiveness is attributed not only to their UV screening properties but also to their long-term protective effects against free radical-induced skin damage. These compounds are absorbed into the body and accumulate in the skin, providing sustained protection against UV radiation^10^. Inorganic sunscreens function by scattering, absorbing and reflecting UV radiation, thereby preventing its penetration into the skin^11^. These sunscreens create a physical barrier against UV and visible light. The primary inorganic UV filters include zinc oxide (ZnO) and titanium dioxide (TiO₂), which are widely utilized in both cosmetic and pharmaceutical formulations^12^.

Organic UV filters, such as benzophenones, function by absorbing UV radiation and transitioning to a higher energy^13^. These compounds typically contain aromatic structures linked to a carbonyl group. Organic UV filters are broadly classified into three categories based on their protective range: UVB (290–320 nm), UVA (320–400 nm), and broad-spectrum sunscreens that cover the entire UV range (290–400 nm)^14^. However, certain organic filters, including para-aminobenzoic acid (PABA) and benzophenones, have been associated with adverse effects such as eczematous dermatitis, skin irritations, a burning sensation, and an increased risk of skin cancer^15^. Following the discovery of skin irritation caused by PABA, subsequent research focused on developing alternative organic compounds, including cinnamates, salicylates, benzophenones, benzimidazoles, and other chromophores^16^. One prominent chromophore is chalcone, which consists of an aromatic ring structure linked to a carbonyl group. Several studies have shown that chalcone derived compounds are capable of absorbing light in the UV region^17^. Chalcone itself exhibits two significant absorption bands: one in the wavelength range of 220–270 nm and another between 340 and 390 nm^18^. The absorption properties of chalcone are associated with electronic transitions in the n → π* and π → π* regions, underscoring its potential as an anti-UV material^19^. Based on these findings, it is hypothesized that structural equivalents of chalcone may exhibit comparable UV-absorbing capabilities. One such similarity is dibenzalacetone (DBA), which shares key structural features with chalcone^20^.

DBA is an organic compound with the molecular formula C₁₇H₁₆O and a molecular weight of 236.31 g/mol. Its IUPAC name is 1,3-diphenyl-2-propanone^21^. It exhibits low solubility in water but is highly soluble in several organic solvents, including ethanol, chloroform, ether, and benzene^22^. Its significant partition coefficient (Log P) reflects its pronounced lipophilicity. Chemically, DBA is stable under normal conditions but is reactive with strong oxidizing agents. Sunscreen formulations are developed in various forms to maximize UV protection, stability, and user compliance^23^.

Despite its promising photoprotective properties, the toxicological profile of DBA remains relatively limited compared with currently approved UV filters. Nevertheless, available studies indicate no clear evidence of carcinogenicity in humans. In addition, DBA and its derivatives have been reported to exhibit several biological activities, including antioxidant^24^, anti-inflammatory^25^, and potential anticancer effects^26^. These findings suggest that DBA may represent a promising candidate for topical photoprotective formulations; however, further toxicological and dermatological evaluations are still required to fully establish its safety profile. Traditional formulations, such as creams, lotions, gels, and sprays, are primarily emulsions consisting of aqueous and lipid phases. Recently, alternative delivery systems like oleogels have gained attention due to their unique lipid based structure^27^.

Oleogels are thermostable in nature; this may be attributed to the ability of the gelators to undergo self-assembly, under suitable conditions resulting in the decrease of the total free energy of the system and renders it as low energy thermostable system^28^. Depending on the composition of the oleogels, the oleogels may be transparent or opaque in nature^29^. In pharmaceutical applications, they are utilized for their inherent thermostability, which contributes to their formulation stability and extended usability. It is also noteworthy that, they have been proposed as delivery systems for bioactive agents, particularly in cases where prolonged shelf-life and sustained release are required^30^.

Another line of evidence shows that, the compatibility of the oil based vehicle with the stratum corneum (SC), along with its favorable texture, consistency, and rheological properties, makes oleogels well-suited for topical application^31^. An essential condition for an ideal oleogel is good spreadability, which refers to the extent to which the gel readily disperses upon application to the skin or the targeted area, ensuring uniform coverage and effective sunscreen delivery^32^. Due to their oleophilic nature, oleogels exhibit limited washability from the skin. The degree of water resistance directly influences the duration of skin retention, allowing for prolonged therapeutic or protective effects^33^.

Despite the recognized photoprotective potential of DBA, its practical application in sunscreen formulations remains limited due to its poor aqueous solubility, lack of structured delivery systems, and insufficient exploration within advanced lipid-based matrices. Moreover, current oleogel-based photoprotective systems predominantly rely on conventional gelators without fully exploiting the structural and functional advantages of particulate gel networks. To date, no studies have reported the incorporation of DBA into silicon dioxide (SD)-based oleogels as a structured photoprotective platform. The use of SD as a particulate oleogelator offers the dual advantage of forming a stable three-dimensional network while potentially enhancing UV scattering. In addition, the integration of biologically active oils such as argan oil (AO), rich in tocopherols and polyphenols, and jojoba oil (JO), known for its skin-mimetic and biocompatible properties, may provide synergistic antioxidant, photoprotective, and dermal compatibility benefits. Therefore, the present work introduces a structured SD-based oleogel system integrating functional oils as an innovative strategy to enhance the stability, efficacy, and topical performance of DBA in sunscreen formulations. This study aims to develop and evaluate oleogels as cosmetic bases for the incorporation of DBA. A factorial design will be employed to investigate the influence of three independent variables oil type, oleogelator concentration and drug concentration on the physical stability of the formulated oleogels.

## Materials and methods

### Materials

Dibenzalacetone (DBA) was synthesized and prepared in the laboratory of the Faculty of Pharmacy, October 6 University. Jojoba oil (JO), argan oil (AO), and black seed oil (BS), all analytical grade, were utilized without further purification. Ethanol and methylene chloride (HPLC grade), along with disodium hydrogen phosphate, potassium chloride, Tween 80, potassium dihydrogen phosphate, silicon dioxide (SD) and sodium chloride, were procured from El-Nasr Pharmaceutical Chemical Co., Egypt. A cellulose membrane with a molecular weight cut-off of 12,000 Daltons was obtained from Sigma-Aldrich (USA). Additional materials included double-distilled water, helioscreen plates (North Sutton, NH, USA), and excised newborn rat skin, which was supplied by the animal house at Cairo University, Egypt. Human keratinocyte (HaCaT) cell line was obtained from the American Type Culture Collection (ATCC, Manassas, VA, USA) and cultured under standard conditions.

### Methods

#### Synthesis of dibenzalacetone (DBA) *via* the Claisen–Schmidt Reaction

**Fig. 2:**
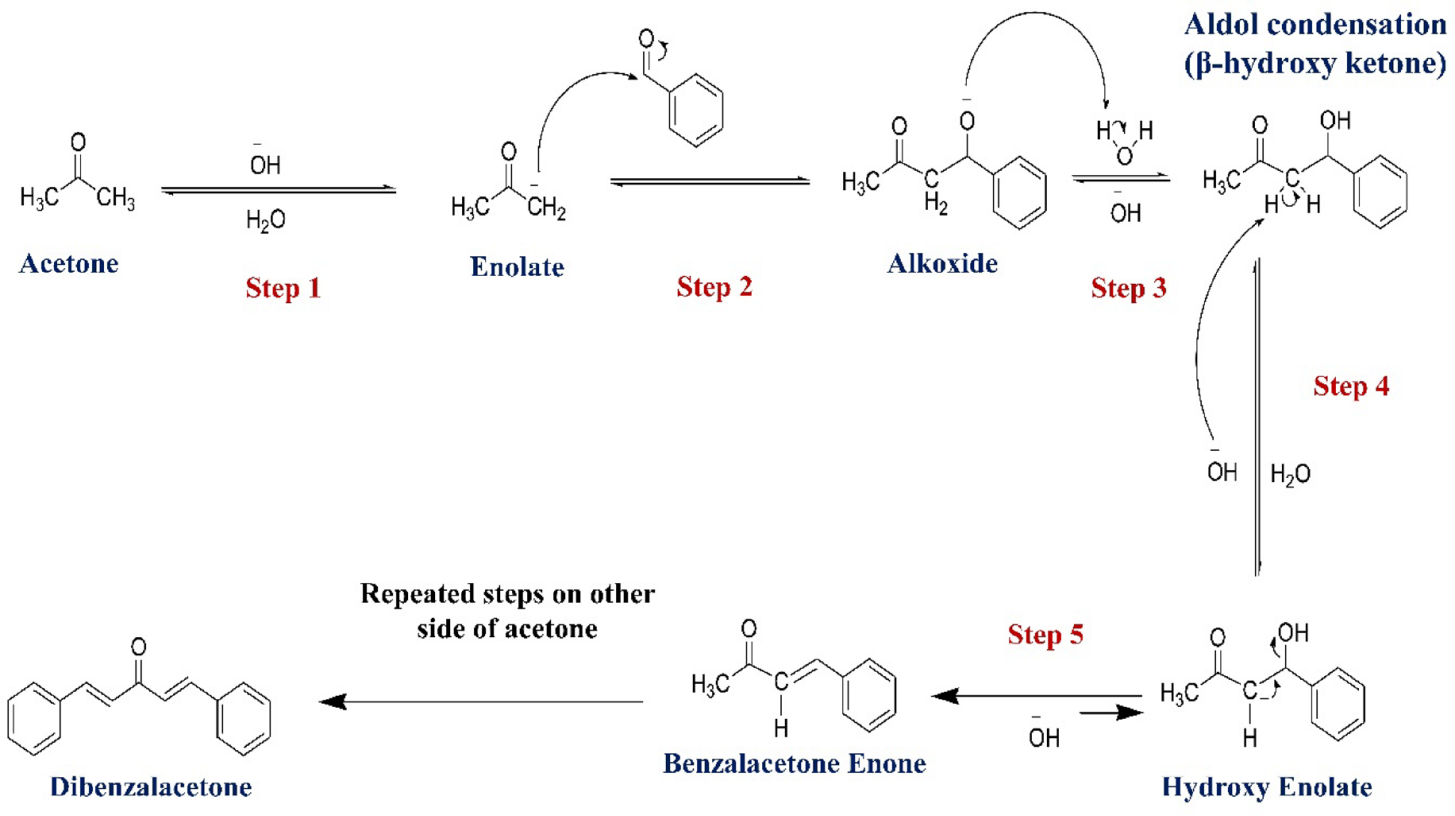
Synthesis of Dibenzalacetone (DBA) *via* the Claisen–Schmidt Reaction^24^.

The synthesis of DBA typically occurs *via* a cross-aldol condensation, specifically the Claisen-Schmidt reaction, which involves benzaldehyde and acetone as illustrated in the mechanism presented in (Fig. 2). Acetone, which contains alpha H atoms, reacts with benzaldehyde, which misses these hydrogens. An ethanolic base facilitates the process by reacting with NaOH to produce sodium ethoxide **(**CH_3_CH_2_ONa). Sodium ethoxide abstracts the alpha hydrogen from acetone, generating an enolate ion. This enolate ion, acting as a nucleophile, donates electrons and attacks the carbonyl group of benzaldehyde. The resulting product is a β-hydroxy ketone or β-hydroxy aldehyde, known as an aldol. This intermediate then undergoes dehydration, eliminating a water molecule and forming DBA^24^.

#### Preformulation studies

To identify a suitable oil phase for the formulation of oleogels, the solubility of DBA was assessed in various oils, specifically Argan oil (AO), Jojoba oil (JO), and another oil referred to as black seed oil (BS), following the methodology described by Sheta et al.^34^. An excess quantity of DBA was introduced into 5 mL of each oil in stoppered glass vials and subjected to continuous agitation at 25 ± 0.5 °C for 72 h to attain equilibrium. Post-equilibration, the mixtures were centrifuged at 2500 rpm for 30 min to remove any undissolved drug. The resulting supernatants were subsequently filtered using a 0.45 μm Millipore membrane filter, and the concentration of DBA in the filtrate was quantified spectrophotometrically at its predetermined λ max after appropriate dilution with methylene chloride^35^.

#### Determination of critical gelation concentration (CGC)

The critical gelation concentration (CGC) is defined as the minimum concentration of an oleogelator required to induce gelation of the oil phase^34^. To determine the CGC, oleogels were prepared using increasing concentrations of the oleogelator silicon dioxide (SD), ranging from 2.5 to 15% w/w, with incremental increases of 2.5% w/w, in selected oils, specifically AO and JO, as outlined by Motulsky et al.^36^. Each formulation was prepared by accurately weighing the oleogelator and incorporating it into the oil within a test tube. The mixtures were then heated to 70 °C and stirred manually with a glass rod until a transparent and homogeneous solution was obtained^37^. The samples were subsequently allowed to cool to 25 °C and were inverted to assess gel formation. This procedure was systematically repeated for each concentration to establish the CGC for the respective oil–oleogelator combination^38^.

#### Experimental plan of the factorial design used for the preparation of DBA oleogel formulae

A full factorial design (2³) was employed for the formulation of DBA loaded oleogels to systematically evaluate the effect of three independent variables, as detailed in Table 1. The variables included: (X_1_) oil type, assessed at two levels AO and JO; (X_2_) oleogelator concentration, examined at 10% and 15% w/w; and (X_3_) drug concentration, investigated at 4% and 8% w/w. This design enabled the assessment of both individual and interactive effects of these factors on the formulation characteristics.

**Table 1:** Experimental plan of the factorial design (2^3^) used for the preparation of DBA oleogel formulae.

Variables	Levels
X1: Oil type	Argan Oil (AO)
	Jojoba Oil (JO)
X2: Oleogelator concentration (Silicon dioxide (SD))	10% w/w
	15% w/w
X3: Drug concentration (DBA conc.)	4% w/w
	8% w/w

#### Preparation of DBA oleogel formulae

The composition of the various oleogel formulations prepared according to the factorial design parameters is detailed in Table 2. Each formulation was prepared by incorporating the oleogelator into the selected oil, followed by heating the mixture to a temperature slightly above the oleogelator’s melting point (not exceeding 80 °C), with continuous stirring to ensure complete dispersion, as previously described by^39^. Once a clear, homogeneous solution (sol state) was achieved, the designated amount of DBA was incorporated. The mixture was then removed from the heat source and stirred continuously until it reached ambient temperature. The prepared oleogels were left undisturbed at room temperature for 48 h to facilitate physical stabilization and structural organization. During this period, the system underwent a sol-to-gel transition, with crystal formation contributing to the development of a three-dimensional network structure throughout the oil phase (gel state)^40^.

**Table 2:** The results of DBA loaded oleogel formulae of the experimental full (2^3^) factorial design.

Formula Code	X1 Oil types	X2 SD Concentration (w/w%)	X3 DBA Concentration (w/w%)	Y1 SPF	Y2 Sunscreen efficacy
F1	JO	10	4	6.6±0.14	0.45±0.02
F2	JO	10	8	12.45±0.07	0.31±0.01
F3	JO	15	4	11.05±0.21	0.395±0.007
F4	JO	15	8	14.05±0.08	0.265±0.007
F5	AO	10	4	7.8±0.14	0.37±0.02
F6	AO	10	8	13.63±0.01	0.23±0.00
F7	AO	15	4	10.63±0.18	0.27±0.007
F8	AO	15	8	18.63±0.64	0.22±0.02

AO, argan oil; DBA, dibenzalacetone; SD, silicon dioxide; JO, jojoba oil; SPF, sun protection factor.

Data are presented as mean ± SD (*n*=3).

#### Evaluation tests of the different prepared DBA oleogel formulae

##### Organoleptic properties of DBA oleogel formulae

The prepared DBA oleogel formulations were subjected to a series of visual and organoleptic evaluations to assess their physicochemical characteristics. Parameters such as color, clarity, fluidity, and phase separation were examined. Alongside these observations, organoleptic attributes including texture, consistency, and homogeneity were evaluated as described by Jha S and Maurya^41^. Each formulation was also inspected for the presence of particulate clogs and any abrupt changes in viscosity. To assess physical properties, a small amount of each formulation was applied topically, and the sensory experience upon application was documented^42^.

##### Non-birefringence examination under cross-polarized microscope

The DBA oleogel formulations were examined using cross-polarized light microscopy to assess the presence or absence of birefringence, thereby excluding the formation of liquid crystalline structures, as reported by Jha and Maurya^43^. A small aliquot of each sample was carefully placed on a clean glass slide and analyzed under a cross-polarized microscope to determine the optical anisotropy of the system.

##### Physical stability of DBA oleogel formulae

Physical stability provided useful information over a short period of time^44^. To evaluate the physical stability of the various prepared DBA oleogel formulae, three tests were performed.

a. Centrifuge stress test

The DBA oleogel formulations were subjected to centrifugation at 3000 rpm for 30 min to evaluate their physical stability, specifically for signs of liquefaction and phase separation. Formulations that remained intact without any visible phase separation were subsequently selected for further evaluation through thermal stress testing using the cooling–heating cycle method^44^.

b. Cooling-heating cycle

The DBA oleogel formulations were subjected to three complete thermal cycles, each comprising 24 h at 5 °C followed by 24 h at 45 °C. This procedure was employed to evaluate the formulations resistance to temperature fluctuations, simulating environmental storage conditions. Formulations that remained physically stable without exhibiting phase separation were subsequently advanced for freeze thaw stress testing to further assess their strength under extreme thermal conditions^45^.

c. Freeze thaw stress test

The DBA oleogel formulations were subjected to three consecutive freeze–thaw cycles, with each cycle comprising 24 h at−5 °C followed by 24 h at 25 °C. This protocol was designed to evaluate the formulations resistance to thermal stress and assess their physical stability under fluctuating temperature conditions, as described by Sagiri et al.^46^.

##### Rheological properties measurements

The rheological properties of the prepared oleogels were evaluated using a Brookfield digital viscometer (RV-TD)^47^. Each formulation (25 g) was subjected to varying rotational speeds (0.5–100 rpm) under ambient conditions, with shear stress plotted against shear rate^48^. Farrow’s constant (N) was determined to assess pseudo-plasticity, while the hysteresis area (H.A) was calculated to characterize thixotropic behavior. Flow properties were analyzed using the equation:

1 $$\:\mathrm{l}\mathrm{o}\mathrm{g}\:\mathrm{S}\:=\:\mathrm{N}\:\mathrm{l}\mathrm{o}\mathrm{g}\:\mathrm{D}\:-\:\mathrm{l}\mathrm{o}\mathrm{g}\:{\upeta\:}$$

where N values greater than 1 indicated pseudo-plastic flow, and values less than 1 suggested dilatant behavior. Viscosity was measured at both minimum (ηmin) and maximum (ηmax) shear rates^49^.

##### Drug content

A 0.1 g of the each selected DBA oleogel formulae was dissolved in a 100 mL volumetric flask containing methylene chloride^42^. This solution was left for 15 min with continuous stirring using a magnetic stirrer to ensure that the drug was completely dissolved. The absorbance of this solution was measured spectrophotometrically at the predetermined λ-max of DBA and the percentage of drug content in each oleogel was calculated using the following equation^40^:

2 $$\:Percentage\:of\:DBA\:in\:oleogel=\frac{\mathrm{C}\mathrm{o}\mathrm{n}\mathrm{c}\mathrm{e}\mathrm{n}\mathrm{t}\mathrm{r}\mathrm{a}\mathrm{t}\mathrm{i}\mathrm{o}\mathrm{n}\:\mathrm{o}\mathrm{f}\:\mathrm{D}\mathrm{B}\mathrm{A}\:\:\mathrm{i}\mathrm{n}\:\mathrm{o}\mathrm{l}\mathrm{e}\mathrm{o}\mathrm{g}\mathrm{e}\mathrm{l}}{\mathrm{C}\mathrm{a}\mathrm{l}\mathrm{c}\mathrm{u}\mathrm{l}\mathrm{a}\mathrm{t}\mathrm{e}\mathrm{d}\:\mathrm{c}\mathrm{o}\mathrm{n}\mathrm{c}\mathrm{e}\mathrm{n}\mathrm{t}\mathrm{r}\mathrm{a}\mathrm{t}\mathrm{i}\mathrm{o}\mathrm{n}\:\mathrm{o}\mathrm{f}\:\:\mathrm{D}\mathrm{B}\mathrm{A}\:\:}\:\times\:100$$

##### Test for spreadability

Spreadability of DBA oleogel was measured in terms of the diameter of a circle produced when a weighed quantity (0.5 g) of DBA oleogel was placed between two glass plates and pressed by the known weight (1 kg) left for about 5 min where no more spreading was expected^50^. The diameter of the formed circle was measured and taken as comparative value for spreadability^51^. Spreadability of the tested bases was measured in terms of the average diameter of the spreaded circle and compared to the spreadability of Lassar’s paste of low spreadability (diameter = 2.6 cm), hydrophilic ointment (USPXXII) of medium spreadability (diameter = 4.0 cm), and aqueous cream of high spreadability (diameter = 5.0 cm)^51^.

##### Determination of gel-sol transition temperature (*Tg*) by simple test tube inversion method (Thermoreversibility)

The prepared DBA oleogel (5 g) was subjected to increasing temperatures starting from 30 to 80 °C^52^. An increment of 5 °C increase in temperature was made after 5 min incubation at the previous temperature. The temperature, at which the gels started to flow, when the test tubes were inverted and this, was noted as the *Tg*^53^. The guideline for this technique was suggested by Sheta et al.^54^.

##### In vitro measurement of sun protection factor

The measurement of SPF value for each formula was done and calculated using a Labsphere UV 2000 S ultraviolet transmittance analyzer (Labsphere, Inc., North Sutton, NH USA) at wavelength range 290–400 nm^19^ through a sample placed on a PMMA plate with the selected formula. Weigh and apply 2 mg/cm^2^ of each formula over the PMMA plates which possess topography parameters totally in compliance with ISO 24,443 method–Annex D method and to the Colipa rev. 2011 method. The application of each formula was done by the aid of micropipette and rubs it with a single finger-cot-coated then put the plate aside to dry for at least 20 min before measurements begin. At least three samples were prepared for each formula. To ensure reproducibility and accuracy in SPF determination, a standard sunscreen formulation was prepared according to the FDA and Australian/New Zealand sunscreen evaluation guidelines. The reported SPF value of this standard formulation is 4.47 ± 1.279^55^.

##### Sunscreen efficacy testing

A 0.05% w/w solution of sodium nitroprusside in distilled water was prepared and each 40 mL of this solution was placed in three petriplates^56^. One petriplate containing sodium nitroprusside solution was exposed directly to UV lamp (CUV light) and one served as a negative (non-irradiated) control and was kept in a dark place for comparison (C dark), then an aliquot of 2 mg/cm^2^ of DBA oleogel was spread uniformly over the third petriplate as a layer, the first and third petriplates were exposed to UV lamp for 2 hr. After exposure to UV lamp, sodium nitroprusside solutions were analyzed using the spectrophotometric measurements to determine the stability of sodium nitroprusside with most emphasis on increase in the absorbance at 395 nm with degradation. A comparative study was carried out to determine the highest protection of the prepared DBA oleogel confirmed by the lower blue color intensity formation^56^.

##### In-vitro permeation studies of DBA through excised newborn rat skin

- Preparation of full excised newborn rat barrier membrane

The full body skin of newborn rat (one week aged hairless rat) was excised and used. The animals were euthanized by ether overdose and the whole thickness of the abdominal skin was separated from the underlying connective tissue using a scalpel^57^. The whole thickness of the skin was used in diffusion experiments. The subcutaneous tissue was removed surgically if present and the dermis side was wiped with isopropyl alcohol to remove adhering fat. The cleaned skin was washed with distilled water and stored in the deep freezer until further use. The skin was brought to room temperature when used^58^.

b. In vitro permeation test

In vitro permeation of DBA from the selected DBA oleogel formulae was performed using a USP XXXI dissolution apparatus II (rotating paddle) with slight modification: a 0.5 g of the selected oleogel formulae were spread over the surface of a glass slide of 8.03 cm^2^ surface area and then covered with the newborn rat skin where the stratum corneum side was opposite downward into the oleogel and the dermal side was opposite upward into the receiver medium. The glass and the excised rat skin were held together by water proof plaster and equally spaced plastic clips^51^. This assembly was placed at the bottom of the dissolution vessel containing 300 mL of PBS (pH = 7.4) containing 1% Tween 80 (w/v)^56^. 1% (w/v) Tween 80 was added into the dissolution medium to maintain sink condition^59^. The amount of DBA permeated was determined for 8 h at 32 ± 1 °C at a speed of 100 rpm. Aliquots of 5 mL were withdrawn every 60 min for 8 h; each was replaced with equal volume of fresh medium to maintain a constant volume. The concentration of DBA was determined spectrophotometrically against appropriate blank at the predetermined λ _max_^60^.

##### Statistical analysis

All data are presented as mean ± standard deviation. Statistical analysis was performed using one-way analysis of variance (ANOVA), followed by Tukey’s post-hoc test for multiple comparisons (*n* = 3) using Graphpad^®^ Instat software (version 3.06, GraphPad Software, Inc., La Jolla, CA, USA). A *P-*value < 0.05 was considered statistically significant^61^.

#### Evaluation tests of the optimum DBA oleogel formula

##### Water resistant test

The water-resistance component of the SPF test involved alternating cycles of fresh water immersion and drying. Sunscreen formulations were applied to helioscreen plates, which were subsequently immersed in a shaking water bath containing 5 L of fresh water maintained at 23–32 °C. The bath was agitated at 30 rpm to simulate moderate activity and to avoid excessive accumulation of dispersed sunscreen that could lead to re-adsorption^62^. Each immersion cycle was followed by a 20 min air-drying period, with no drying, until the total water exposure time was achieved. SPF values were measured before and after each immersion cycle to determine the degree of protection retained following water exposure^63^. According to standardized criteria, a formulation is considered water resistant if its percentage water resistance retention (%WRR) exceeds 50%, and very water resistant (or waterproof) if the %WRR exceeds 80% after two consecutive 20 min immersion cycles.

##### Differential scanning calorimetry (DSC)

Differential scanning calorimetry study was done to the optimum DBA oleogel formula, to investigate the crystalline status and the extent of DBA within these oleogel formula^64^. About 2–4 mg of DBA, oleogelators (SD), plain oleogel base as well as DBA oleogel formula were sealed in a 30 µl aluminium pans and heated in the DSC instrument under dynamic nitrogen atmosphere with the flow rate of 20 mL/min. The temperature range of 25–100 °C was used and the heating rate was 10 °C/min^65^. The DSC thermograms were recorded^50^.

##### Fourier transform infrared spectroscopy (FT-IR)

Infrared spectroscopy was carried out by using FT-IR^66–68^. DBA, plain and loaded with DBA oleogel sample were prepared in a potassium bromide discs and were scanned in the spectral range of 4000 to 400 cm^− 1^ at a resolution of 2 and 50 co-added scans to understand the interactions amongst the components of the oleogel^37^.

##### Cytotoxicity assay of DBA

The cytotoxicity of the optimized DBA-loaded oleogel formulation (F8) on human skin cells was evaluated using the MTT assay. Human keratinocyte (HaCaT) cells were seeded in 96-well plates at a density of 1 × 10⁴ cells/well and incubated overnight at 37 °C in a humidified atmosphere containing 5% CO₂. The cells were subsequently treated with different concentrations of the optimized formulation (F8) (50–800 µg/mL) for 24 h^69^. Untreated cells and vehicle-treated cells were included as controls. Following the exposure period, the cells were incubated with 0.5 mg/mL MTT solution for 3 h. The resulting formazan crystals were dissolved in DMSO, and the absorbance was measured at 570 nm using a microplate reader. Cell viability was expressed as a percentage relative to the control group. All experiments were performed in triplicate, and the results were presented as mean ± standard deviation^70^.

##### Skin irritation test

The skin irritation test was performed to evaluate the potential dermal irritation of the optimized oleogel formulation (F8). Male albino rats weighing 150–180 g were used for the experiment. The dorsal surface of the rats was carefully shaved using electric clippers 24 h prior to the study to remove hair and expose the skin area. The animals were randomly divided into three groups (*n* = 3 per group). The first group served as a control group and received no treatment. The second group received 0.5 mL of 0.8% (v/v) aqueous formalin solution as a standard irritant. The third group received 0.5 g of the optimized oleogel formulation (F8). Each treatment was topically applied to a defined area (approximately 5 cm²) on the shaved dorsal skin once daily for three consecutive days according to the method described by Elmowafy et al.^71^. At the end of the experiment, the animals were euthanized using an overdose of pentobarbital sodium (200 mg/kg, i.p.) in accordance with the guidelines of the Institutional Animal Care and Use Committee (IACUC). Skin samples from both treated and untreated areas were carefully excised for further histopathological examination^72^.

Autopsy skin samples were fixed in 10% neutral buffered formalin for 24 h. The tissues were then washed with tap water and dehydrated using graded ethanol series, followed by clearing in xylene and embedding in paraffin wax. The paraffin blocks were sectioned into 4 μm thick slices using a microtome. The obtained sections were mounted on glass slides, deparaffinized, and stained with hematoxylin and eosin (H&E). The stained sections were examined under a light microscope and compared with the control samples^73^.

## Results and discussion

### Preformulation studies

#### Screening of DBA in different oils used for oleogel preparation

Solubility of DBA in the oil phase is an important condition, as it maintains the presence of the drug in its solubilized form^74^. Therefore, for developing a suitable oleogel system for topical application of DBA, the solubility of DBA in various oils was determined at 25 ± 0.5 °C. Statistical analysis between the solubility of DBA in different oils was computed by one-way analysis of variance (ANOVA) by GraphPad instat Demo at (*p* = 0.05). Results showed that the highest significant (*P* < 0.05) solubility of DBA was found in case of AO (204.3 ± 1.62 mg/mL) and JO (249.4 ± 1.36 mg/mL), while a non-significant difference (*P* > 0.05) was obtained between BS (108.2 ± 0.93 mg/mL). Based on the saturated solubility outcomes AO and JO were chosen as the hydrophobic liquid for the preparation of DBA oleogels.

#### Determination of critical gelation concentration (CGC)

**Fig. 3:**
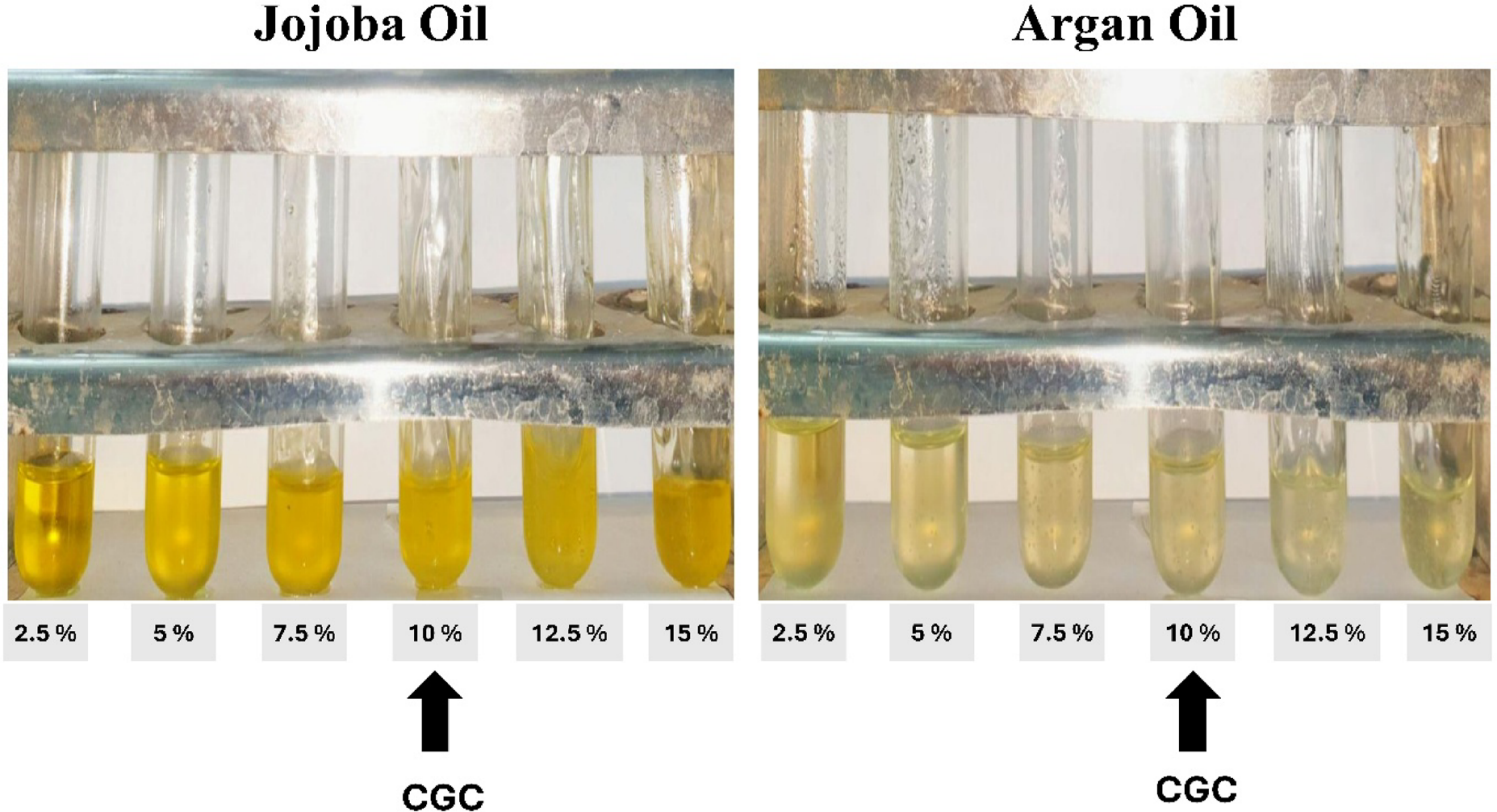
The critical gelation concentration (CGC) of SD at 10% for both JO and AO.

The Critical Gelation Concentration (CGC) assay was conducted to determine the minimum concentration of the oleogelator necessary to induce gelation and ensure the structural stability of the oil-based system, specifically with JO and AO^75^. The results indicated that the CGC of SD in both JO and AO was 10%, as shown in Fig. 3. The CGC value of 10% of SD in both JO and AO indicates that a minimum of 10% oleogelator is required to form a stable three-dimensional gel network capable of immobilizing the oil phase. The identical CGC in both oils suggests that SD exhibits consistent gelation efficiency regardless of the oil type, which may be attributed to similar physicochemical interactions such as hydrogen bonding and *Van der Waals* forces between the silica particles and the lipid components of JO and AO^76^. This finding highlights the versatility of SD as an oleogelator and its potential for application across different oil-based formulations without the need for major concentration adjustments.

### The formulation experimental design

A complete factorial design and statistical analysis were used to determine the effect of the independent variables on the characteristics of DBA loaded oleogel. The studied factors were determined after several experimental trials. The independent variables and observed responses for the F1-F8 formulations were summarized in Table 2. It was observed that the predicted R^2^ values were in good harmony with the adjusted R^2^ values in all examined responses as shown in Table 3. The adequate precision value is for verifying the model’s adequacy to navigate the design space in which a ratio above 4 is suitable, and that was detected in all studied responses (Table 3).

**Table 3:** A statistical investigation summarizes a full (2^3^) factorial design used to optimize DBA loaded oleogel formulae, with the predicted and observed responses of the optimum DBA loaded oleogel formula (F8).

Responses	R2	Adjusted R2	Predicted R2	Adequate precision	Significant factors
Y1: SPF	0.9660	0.9434	0.8926	18.777	X1, X2, X3
Y2: Sunscreen efficacy	0.9580	0.9300	0.8672	17.509	X1, X2, X3
Responses	Y1: SPF	Y2: Sunscreen efficacy			
Observed values	18.63	0.22			
Predicted values	18.0	0.21			

SPF, sun protection factor.

### Evaluation tests of the different prepared DBA oleogel formulae

#### Organoleptic properties of the different prepared DBA oleogel formulae

The Organoleptic properties of the different prepared oleogel formulae varied depending on their composition. The formulated oleogels were inspected visually for color and opacity in addition to odor & texture as shown in Table 4. The physicochemical characteristics of JO and AO, along with the gelation properties of SD, significantly influence the texture, color, and opacity of the resulting oleogels^77^. In terms of texture, oleogels formulated with AO exhibit greater stiff and structural integrity, attributed to the higher viscosity and triglyceride content of AO, which facilitates stronger interactions within the SD gel network^78^. In contrast, JO based oleogels display a softer and smooth consistency due to the lower viscosity and wax ester composition of JO, which results in weaker gelation with SD^79^. The color of these formulations is mainly determined by the intrinsic pigments of the oils, with AO contributing a pale yellow to golden color and JO contributes a light yellow color^80,81^. The presence of SD, a white and inert material, may slightly diminish color intensity due to light-scattering effects within the gel matrix^82^.

**Table 4:** Organoleptic properties of the different prepared DBA oleogel formulae.

Formula	Composition			Texture	Color	Opacity
	Oil Type	SD Conc, (w/w%)	DBA Conc. (w/w%)			
F 1	JO	10%	4%	Smooth	Light yellow	Translucent
F 2	JO	10%	8%	Smooth	Light yellow	Translucent
F 3	JO	15%	4%	Smooth	Light yellow	Translucent
F 4	JO	15%	8%	Smooth	Light yellow	Translucent
F 5	AO	10%	4%	Stiff	Pale yellow to golden	Translucent
F 6	AO	10%	8%	Stiff	Pale yellow to golden	Translucent
F 7	AO	15%	4%	Stiff	Pale yellow to golden	Translucent
F 8	AO	15%	8%	Stiff	Pale yellow to golden	Translucent

AO, argan oil; Conc.: concentration; DBA, dibenzalacetone; SD, silicon dioxide; JO, jojoba oil.

The translucent appearance observed in both AO and JO-based oleogels suggests that the gel networks formed in both systems are not dense enough to completely block light, but are structured sufficiently to scatter some of it, producing a semi-transparent effect^83^. This can be attributed to the formation of a three-dimensional network with medium-sized pores that allow partial light transmission, regardless of the oil type^84^. Additionally, the uniform dispersion of the oleogelator particles in both formulations prevents the formation of highly dense regions or aggregates, thereby maintaining consistent light permeability^85^. The similarity in appearance indicates that, under the same preparation conditions, the influence of oil composition whether rich in wax esters, as in JO, or in fatty acids, as in AO on light scattering properties was minimal^86^. The overall visual and structural characteristics of these oleogels are modulated by key formulation parameters, including SD concentration, particle size, and dispersion efficiency.

#### Non-birefringence examination under cross-polarized microscope

The non-birefringence observed in AO and JO with SD oleogels under cross-polarized light microscopy suggests a lack of crystalline or anisotropic structures within the gel matrix, indicating that the network formation is governed by amorphous colloidal interactions rather than ordered molecular self-assembly^53^. Unlike conventional oleogelators that induce birefringence through fibrillar or lamellar arrangements, SD functions as a particulate oleogelator, forming a three-dimensional network *via* hydrogen bonding and van der Waals forces without generating long-range molecular order^87^. The isotropic nature of these oleogels suggests that structuring relies on physical entrapment of the oil phase within the SD matrix, with no evidence of crystalline domain formation that would contribute to birefringence^32^.

**Fig. 4:**
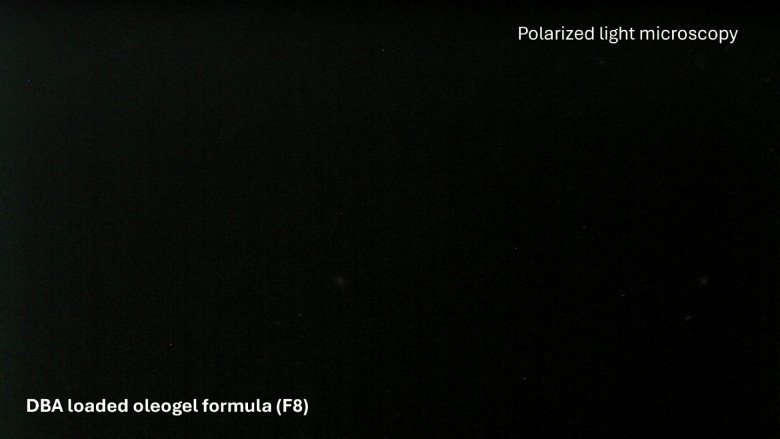
Cross polarized microscopy photograph of (F8) freshly prepared oleogel formula.

The amorphous nature of the network is further supported by the rheological behavior of these systems, particularly in JO based oleogels, where the presence of liquid wax esters results in weaker intermolecular interactions compared to the triglyceride-rich AO-based formulations^88^. These findings confirm that SD acts mostly as a rheological modifier rather than a crystallization-inducing agent, contributing to the viscoelastic and optical properties of the oleogel, which are critical for pharmaceutical and cosmetic applications. DBA Oleogel formulae prepared from SD as gelator (F8) appeared as a dark matrix under polarized light as shown in (Fig. 4). This dark appearance under polarized light confirms the isotropic and non-birefringent nature of the oleogel system^53^.

#### Physical stability of DBA oleogel formulae

All the formulated DBA oleogels were subjected to various stress tests, including centrifugation, cooling-heating cycles, and freeze-thaw cycles, to simulate potential environmental changes during storage, transportation, and application, thereby evaluating their physical stability^44^. Building on these results, SD-based oleogel formulations demonstrated significant stability (*P* < 0.0001) at SD concentrations of 10% and 15% (w/w) upon exposure to stress conditions. Previous studies have highlighted the role of SD as a viscosity enhancing agent in pharmaceutical formulations, contributing to the thickening and thixotropic properties of liquid and semi solid dosage forms although improving their physical stability during storage^37^.

#### Rheological properties measurements

The rheological data (η_min_, η_max_, Farrow’s constant (N) and the hysteresis area (H.A) for all the prepared oleogel formulae are listed in Table 5. The rheological analysis of the DBA-loaded oleogel formulations, as presented in Table 5, confirmed their non-Newtonian, pseudo-plastic flow with thixotropic behavior. The hysteresis area (H.A.) ranged from 0.81 to 8.30 cm², indicating variability in structural recovery. The viscosity of the oleogel systems decreased with increasing shear rates, demonstrating shear-thinning behavior^40^. At lower shear rates, the oleogels exhibited semisolid characteristics due to the physical interactions within the fibrous network. As shear stress increased, these interactions progressively weakened until complete structural disruption allowed the oleogels to flow^89^.

**Table 5:** Rheological parameters of the different freshly prepared DBA oleogel formulae.

Formula	Viscosity at min. rate of shear (cps)	Viscosity at max. rate of shear (cps)	Farrow’s constant *N*	H.A* (cm2)	Flow behavior
F 1	276,000	7512	3.06	1.33	Non - Newtonian pseudo-plastic with thixotropy
F 2	814,90	2419.3	3.47	1.19	
F 3	460,800	105,60	3.53	3.90	
F 4	610,000	243,00	2.35	1.32	
F 5	592,800	115,00	4.31	4.12	
F 6	610,000	215,40	2.82	0.81	
F 7	1,050,700	215,00	3.20	8.30	
F 8	1,255,200	289,10	2.99	7.61	

H.A: The hysteresis area, cps: centipoise, Min.: minimum, Max.: maximum.

Farrow’s constant (N), indicative of pseudo-plasticity, varied from 2.35 to 4.31 across formulations, suggesting different degrees of structural breakdown and recovery. The pseudo-plastic nature of the oleogels was attributed to the gradual rupture of internal gel structures under shear, followed by partial reformation through Brownian motion^51^. Additionally, oleogelator concentration significantly influenced the rheological properties. Formulations containing 15% oleogelator exhibited significantly higher viscosity (*P* < 0.0001) compared to those prepared with 10%. This increase in viscosity is consistent with previous findings, where higher gelling agent concentrations contributed to a denser and more intricate network structure, thereby enhancing gel strength and resistance to deformation^37^.

#### Drug content

The results of drug content analysis confirmed that the incorporated DBA was uniformly distributed within the selected oleogel formulations, indicating a consistent and reproducible preparation method. The calculated percentage of DBA content in the various oleogel formulations ranged from 96.21 ± 0.71% to 102.6 ± 0.13%, as presented in Table 6, which falls within the pharmaceutically acceptable limits. According to regulatory standards for semisolid dosage forms, the drug content should be between 90.0% and 110.0%, with a relative standard deviation (RSD) not exceeding 6%^90^. The obtained results demonstrate satisfactory content uniformity across the formulations, fulfilling the established acceptance criteria for semisolid drug delivery systems^31^.

**Table 6:** Spreadability measurements, drug content and gel-sol transition temperature (Tg) of the selected DBA oleogel formulae.

Formula	Spreadability (cm) (±S.D)	Comment regarding spreadability	Drug content % (±S.D)	*Tg* (°C)
F1	3.72±0.08	Medium	102.5±1.10	55
F2	4.30±0.25	High	101.4±0.45	55
F3	3.81±0.12	Medium	96.21±0.71	55
F4	4.13±0.19	High	99.35±0.88	55
F5	3.72±0.09	Medium	102.1±0.92	48
F6	4.72±0.06	High	99.85±0.15	48
F7	3.64±0.05	Medium	102.2±0.43	48
F8	4.81±0.03	High	102.6±0.13	48

Data are presented as mean average value (±S.D), *n*=3.

#### Spreadability measurement

Spreadability represents a key parameter in evaluating the ease and uniformity of application of topical formulations. It was assessed by measuring the mean diameter of the area over which the oleogel spread. The obtained values were benchmarked against three commonly used semi-solid pharmaceutical preparations Lassar’s paste, hydrophilic ointment, and aqueous cream as referenced by^35^, and presented in Table 6. The spreadability measurements of the selected DBA oleogel formulations ranged from 3.64 ± 0.05 to 4.81 ± 0.03 cm. Among these, formulations F1, F3, F5 and F7 exhibited moderate spreadability, while formulations F2, F4, F6, and F8 demonstrated higher spreadability.

#### Determination of gel-sol transition temperature (Tg) by simple tube inversion method (Thermoreversibility)

The temperature at which the gel started to flow was regarded as gel-to-sol transition temperature^53^ and was recorded as shown in Table 6. The transition temperature, indicating disruption of the three-dimensional oleogel network, was higher in formulations containing JO (approximately 52–55 °C)^91^ compared to those with AO (47–50 °C)^92^, reflecting the influence of oil viscosity and fatty acid composition on gel strength^93^. Upon cooling, all formulations regained their initial gel structure, confirming the thermoreversible nature of the system, which is governed by physical, non-covalent interactions among SD particles^94^. These results suggest that JO based oleogels exhibit greater thermal stability, making them more suitable for applications requiring resistance to temperature fluctuations.

##### In-vitro measurement of sun protection factor (SPF) and Sunscreen efficacy testing of the selected DBA oleogel formulae

**Fig. 5:**
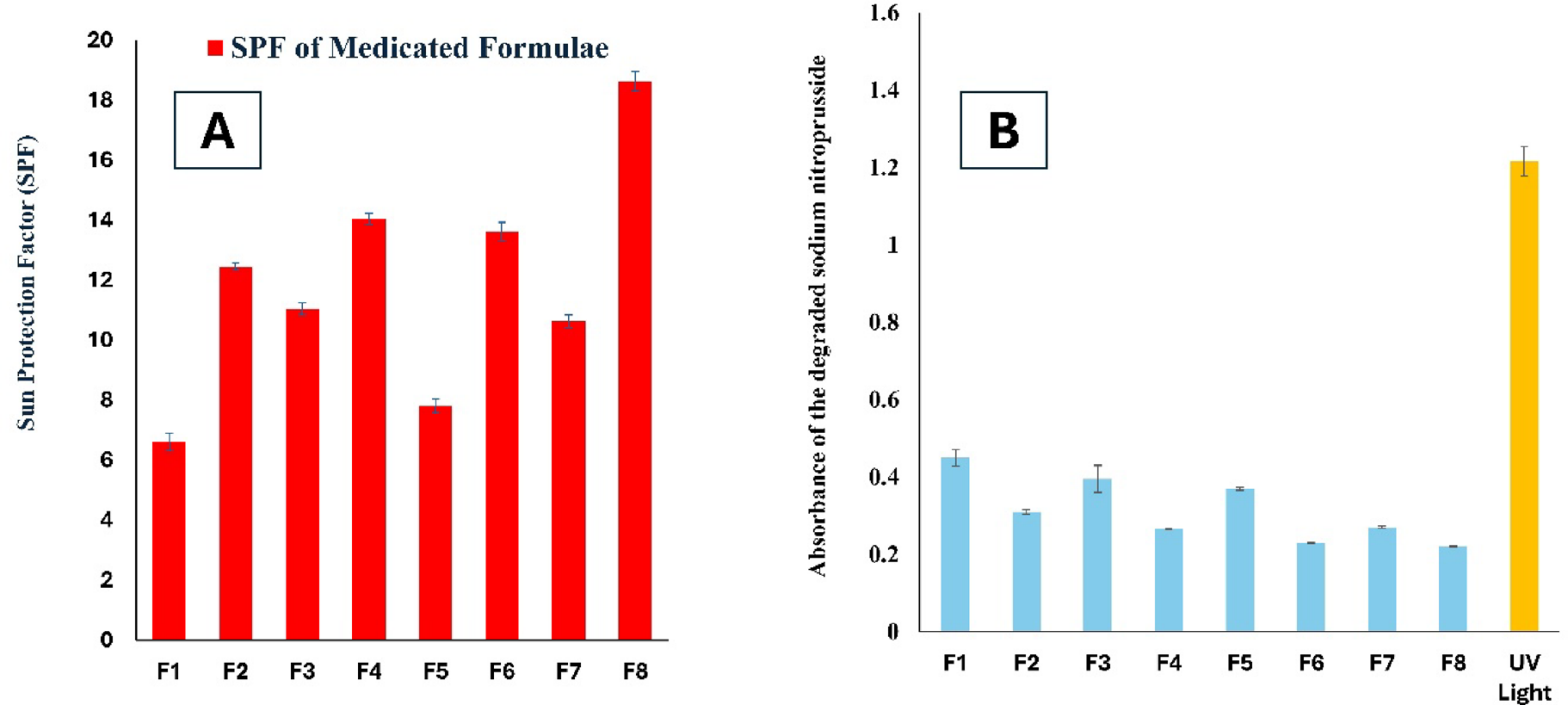
Sun protection factor (**A**) and Sunscreen efficacy testing (**B**) of the medicated oleogel formulae.

**Fig. 6:**
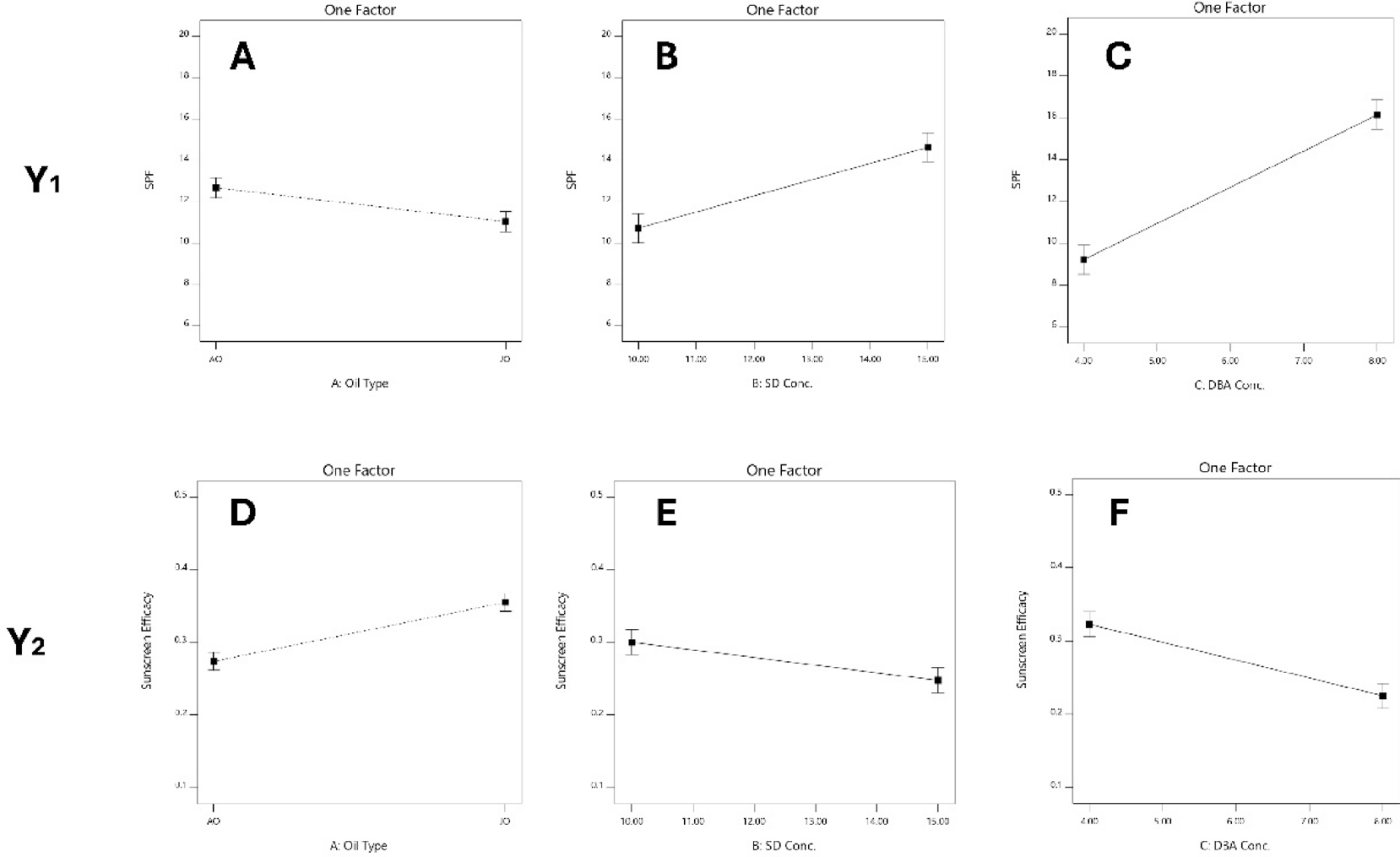
One factor plot of the impact of the amount of oil type (X_1_), SD concentration (X_2_), and DBA concentration (X_3_) on SPF (**A**–**C**), and Sunscreen efficacy (**D**–**F**) of the formulated DBA loaded oleogel. DBA: dibenzalacetone; SPF: sun protection factor; SD: silicon dioxide.

The SPF and sunscreen efficacy values of the prepared DBA-loaded oleogels ranged from 6.60 ± 0.14 to 18.63 ± 0.64 and 0.22 ± 0.02 to 0.45 ± 0.02, respectively, as presented in Table 2 and illustrated in (Fig. 5). Results are revealed as one factor plot in Fig. 6A–F. Figure 6A & D demonstrates that the type of oil (AO or JO) incorporated into the DBA-loaded oleogel (X_1_) had a significant influence on the measured SPF and sunscreen efficacy values (*P* < 0.0045). Specifically, formulations prepared with AO exhibited higher SPF and sunscreen efficacy compared to those prepared with JO. This can be attributed to the superior UV-absorbing, which is rich in tocopherols and polyphenols that provide both direct photoprotection and indirect defence against UV-induced oxidative damage^95^. In contrast, JO, being mainly composed of long-chain wax esters with minimal inherent UV absorption, contributed less to the overall SPF enhancement and sunscreen efficacy^96^. These findings confirm that the choice of oil type is a critical factor in optimizing the sunscreen efficacy of the developed oleogel system.

Considering the effect of SD concentration (X_2_), a significant (*P* < 0.0001) increase in SPF and sunscreen efficacy was observed with the gradual rise in SD content (Fig. 6B & E). This enhancement can be explained by the ability of SD particles to scatter and reflect incident UV radiation, thereby reducing the penetration of harmful rays to the skin surface^16^. In addition, higher SD levels promote better distribution and stabilization of the active sunscreen agent within the oleogel matrix, resulting in a more uniform protective film^97^. However, this positive effect is most pronounced within an optimal concentration range, as excessive amounts of SD may lead to particle aggregation and reduced film uniformity. Overall, the findings confirm that increasing SD concentration plays a crucial role in boosting the sunscreen efficacy of the formulated oleogels.

According to the statistical analysis, the concentration of DBA (X_3_) had a highly significant effect (*P* < 0.0001) on the SPF and sunscreen efficacy of the fabricated oleogels (Fig. 6C & F). Increasing the DBA content led to a observed a rise in SPF and sunscreen efficacy values, which can be attributed to its strong UV-absorbing properties, particularly in the UVB range^98^. A higher amount of DBA enhances the oleogel’s ability to absorb and block harmful UV radiation, thereby improving the overall photoprotective performance^24^.

For the sunscreen efficacy, the absorbance of the degraded sodium nitroprusside solutions (0.05% w/v) protected by the selected oleogel formulae and the control after exposure to UV lamp for 2 h is shown in Fig. 5B. Based on the spectrophotometric analysis, the selected DBA oleogel formulations particularly F8 demonstrated lower absorbance values in comparison to the other tested oleogels and the unprotected control. This reduction in absorbance indicates higher sunscreen efficacy, as these formulations more effectively shielded the sodium nitroprusside solution from photodegradation^99^.

#### In-vitro permeation studies of DBA through excised newborn rat skin

**Fig. 7:**
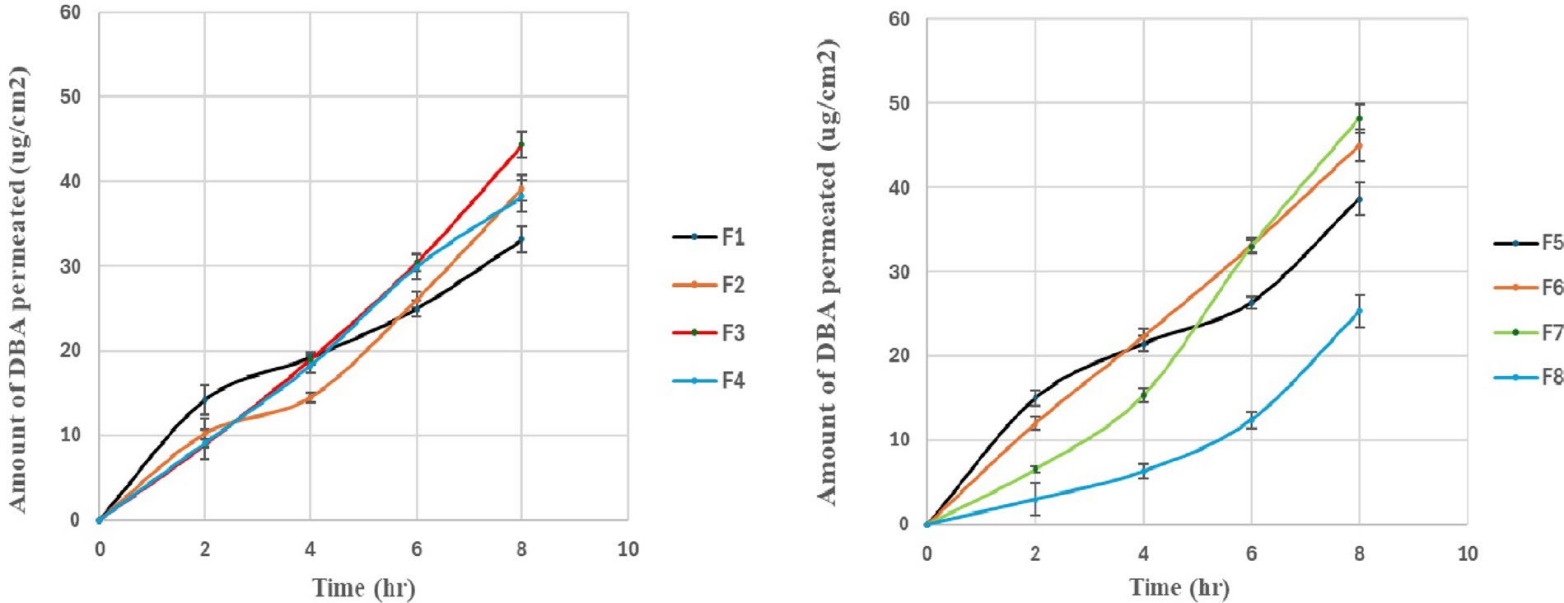
Permeation profiles of DBA through newborn rat skin from the selected oleogel formulae.

The in vitro drug permeation study was performed using excised neonatal rat skin over an 8-hour period (Q₈hr) in Franz diffusion cells containing 300 mL of phosphate-buffered saline (PBS, pH 7.4) with 1% (w/w) Tween 80, maintained at 32 ± 1 °C, following the method outlined by Soliman et al.^51^. This study aimed to evaluate the percutaneous delivery efficiency of eight DBA loaded oleogel formulations. As demonstrated in (Fig. 7), formulation F3 achieved the highest drug permeation, with a significantly greater permeated amount of 109.6 ± 0.23 µg/cm² (*p* < 0.001), followed closely by F5 (108.76 ± 0.36 µg/cm²), F2 (103.24 ± 0.12 µg/cm²), and F7 (99.00 ± 0.61 µg/cm²). Formulations F1 and F6 showed moderate permeation values of 95.2 ± 0.38 µg/cm² and 93.26 ± 0.45 µg/cm², respectively. In contrast, F4 and F8 exhibited the lowest drug permeation levels, recorded at 84.32 ± 0.19 µg/cm² and 83.43 ± 0.53 µg/cm², respectively. These findings indicate that formulation parameters significantly affect DBA transdermal delivery, with F3 demonstrating superior permeation performance among all tested preparations.

The lowest permeated amount was observed with formulation F8, recording 83.43 ± 0.53 µg/cm², which was statistically significant (*p* < 0.001). This limited permeation can be attributed to several factors, including a high affinity of DBA for the oleogel matrix components, reduced thermodynamic activity of the drug within the system, and the hydrophobic nature of the base, which exhibits water resistant properties and elevated viscosity^100^. It is also noteworthy that, the reduced transdermal flux may be explained by the pronounced substantivity of DBA towards keratin, defined as the tendency of a compound to adsorb or absorb onto keratin-rich layers of the epidermis, thereby limiting its diffusion across the skin barrier^101^. The extent of percutaneous drug absorption is administered by multiple parameters, especially the rate of drug release from the vehicle, the physicochemical permeability of the drug through the skin layers, and the rheological characteristics of the carrier^102^. Given that all the evaluated oleogel formulations were characterized by relatively high viscosity, this could have contributed to restricting drug release and subsequent systemic absorption^103^, leading mainly to a localized effect on the skin surface^104^.

### Optimization of DBA loaded oleogel formulation

The Design-Expert^®^ software was utilized to prepare 8 formulae to select the optimum DBA loaded oleogel formula. The main objective of desirability is to predict the optimum levels for the variables under investigation and to assist in selecting the formula of choice. The optimum DBA loaded oleogel formula was F8 which achieving maximum SPF and maximum sunscreen efficacy. F8 was prepared with a desirability = 0.937.

### Evaluation tests for the optimum DBA oleogel formula

Selection of the optimum oleogel formula was carried out according to the highest spreadabilty, highest SPF and highest sunscreen efficacy. This formula was subjected to further tests and storage to assure their stability. The formula which showed these requirements was F8.

#### Water resistant test

The in vitro water-resistance evaluation of the optimized DBA-loaded oleogel formulation (F8) revealed complete removal from the helioscreen plates following the initial 40 min immersion. Accordingly, comparable outcomes were obtained after both 40 and 80 min exposures, indicating that F8 does not exhibit water-resistant properties.

#### Differential scanning calorimetry (DSC)

**Fig. 8:**
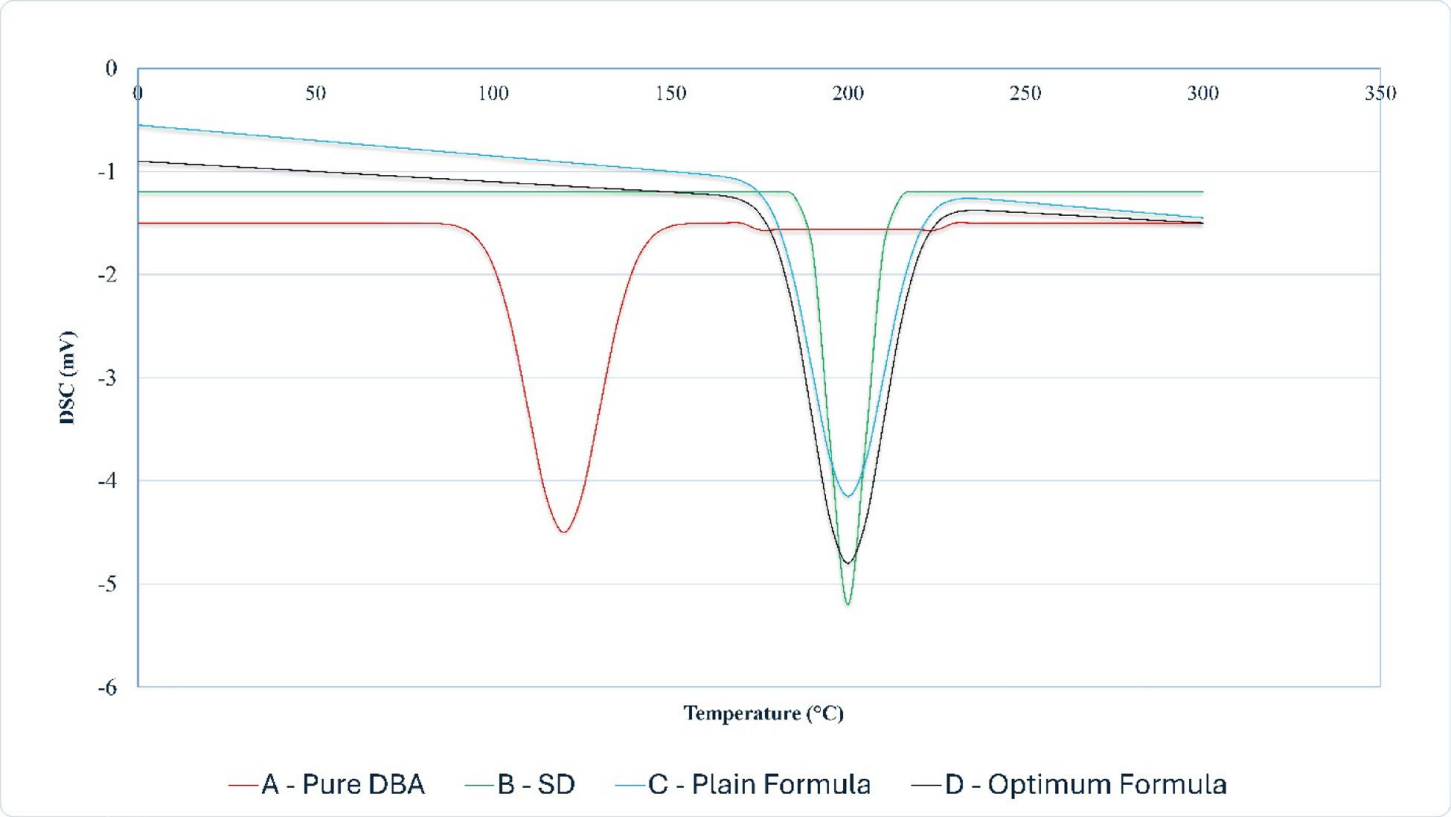
Differential scanning calorimetry thermograms of (**A**) pure DBA, (**B**) SD, (**C**) plain oleogel and (**D**) F8 oleogel.

Differential Scanning Calorimetry (DSC) was employed to assess the melting behavior and crystalline state of DBA, providing insight into its molecular arrangement and physical state within the optimized formulation^64^. The DSC thermograms presented in (Fig. 8), illustrate the thermal profiles of the pure components (DBA and SD), and the corresponding plain oleogel bases. The thermogram of pure DBA exhibited a sharp endothermic peak at 120 °C, corresponding to its melting point, whereas SD demonstrated a maximum endothermic transition at 200 °C. Notably, the DSC profile of the optimized DBA oleogel formulation (F8) revealed the complete disappearance of the characteristic DBA melting peak. This observation suggests a transformation of DBA from its crystalline state to an amorphous or molecularly dispersed form within the oleogel matrix^105^. The absence of a distinct melting peak could also be attributed to the dissolution of DBA within the oil phase, which becomes immobilized within the gelator network, thereby preventing the formation of detectable crystalline structures^106^.

#### Fourier transform infrared spectroscopy (FT-IR)

**Fig. 9:**
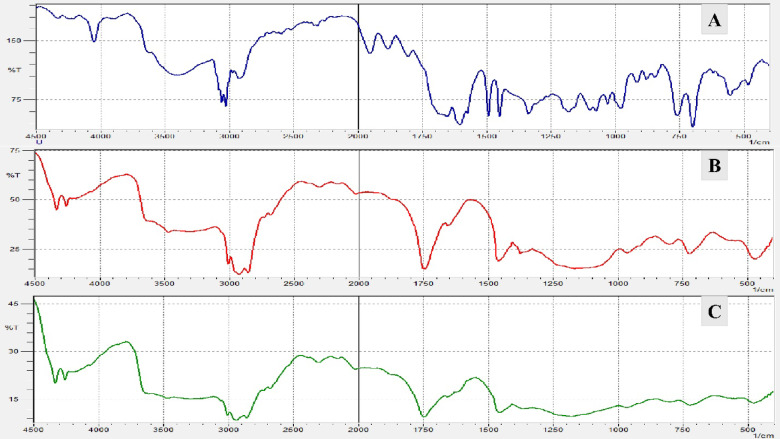
FTIR spectra of (**A**) drug, (**B**) plain F8 (**C**) F8 oleogel.

Fourier Transform Infrared (FTIR) spectroscopy was utilized to investigate potential molecular interactions among the components in the optimized DBA oleogel formulation^107^. The infrared spectra of pure DBA, the plain oleogel bases, and the corresponding DBA oleogel formulation (F8) are presented in (Fig. 9**)**. The FTIR spectrum of DBA exhibited characteristic absorption bands at 3059.1 cm⁻¹ and 3026.31 cm⁻¹, corresponding to C–H stretching vibrations in the benzyl ring. The conjugated system of the compound was further confirmed by the presence of C = C stretching vibrations within the range of 1446–1610 cm⁻¹. Notably, a distinct absorption peak at 1604.77 cm⁻¹ was indicative of the stretching vibration of the carbonyl group (C = O)^108^.

The spectral analysis revealed that all characteristic bands of the plain oleogel base and the corresponding DBA oleogel formulation appeared at identical wavenumbers, suggesting the absence of significant molecular interactions between DBA and the excipients. In parallel, the absorption peaks of the DBA oleogel formulation were more intense than those observed for the plain oleogel, which could be attributed to the incorporation of DBA within the oleogel matrix. These findings align with previous studies reporting similar spectral patterns for drug-loaded gel formulations^109^.

#### Effect of storage on the optimum DBA oleogel formulae

After six months of storage under ambient temperature and humidity conditions, the optimized DBA oleogel formulation (F8) demonstrated physical stability, with no observable alterations in its organoleptic properties, including odor, color, texture, and opacity. Building upon this, examination under a cross-polarized microscope revealed no significant structural changes in the oleogel samples. Regarding drug content, spreadability, in vitro SPF assessment, and sunscreen efficacy testing, no statistically significant differences were observed between the freshly prepared and aged oleogel formulations, confirming their stability over the storage period, as detailed in Table 7. The drug content in the aged oleogel formulation remained within the acceptable variation range of 95–105%^110^, with a relative standard deviation (RSD) not exceeding 6%, meeting the acceptance criteria for semisolid content uniformity^31^.

**Table 7:** Effect of storage of the optimum F8 oleogel formula.

Parameter		F8 Fresh	F8 Aged
Organoleptic properties	Odor	Non-characteristic oily odor and no phase separation	
	Texture	Smooth	Smooth
	Color	Pale yellow to golden	Pale yellow to golden
	Opacity	Translucent	Translucent
Drug content (%)		102.6±0.13	104.2±0.21
Spreadability (cm2)		4.81±0.03	4.77±0.02
SPF		18.63±0.64	18.02±0.48
Sunscreen efficacy testing		0.22±0.02	0.21±0.012

Data are presented as mean average value (±S.D), *n*=3.

**Fig. 10:**
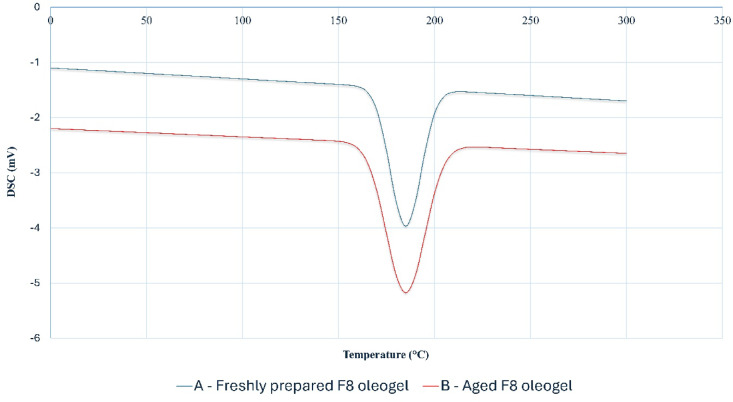
Differential scanning calorimetray thermograms of (**A**) Freshly prepared F8 oleogel and (**B**) Aged F8 oleogel.

Beyond that, spreadability values exhibited no significant difference (*p* > 0.05) between the fresh and stored formulation. Alongside these observations, SPF measurements and sunscreen efficacy assessments, conducted before and after storage, indicated a non-significant difference (*p* > 0.05), further supporting the stability of the formulation, as shown in Table 7. DSC analysis revealed no changes in the thermograms of the stored oleogel formulation, suggesting that the drug remained in a solubilized state within the oleogel matrix throughout the storage period, as illustrated in (Fig. 10). These findings collectively affirm the long-term stability of the DBA oleogel formulation, ensuring its consistency in performance and physicochemical properties.

#### Cytotoxicity assay

**Fig. 11:**
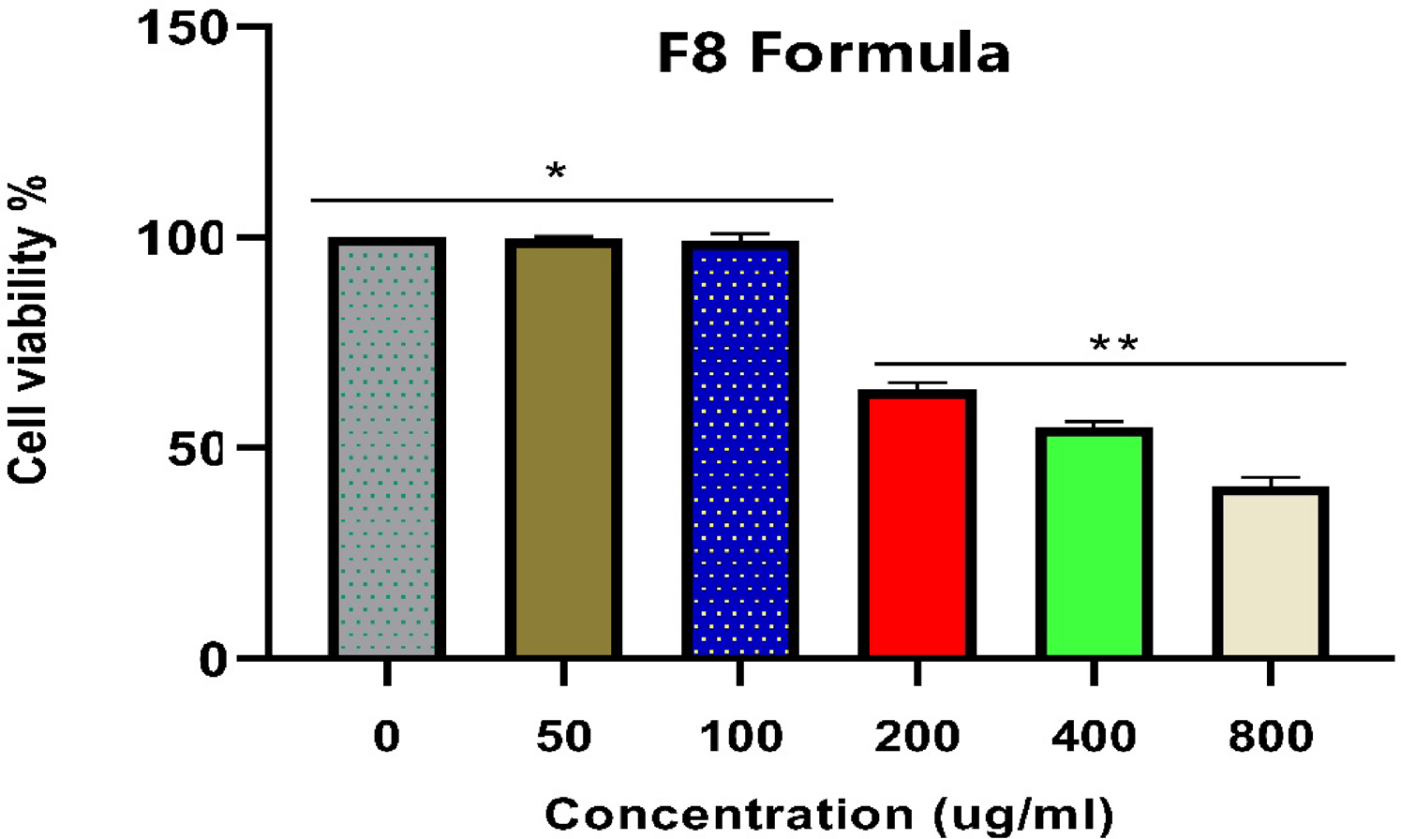
Cell viability of human keratinocyte (HaCaT) cells after 48 h incubation with the optimized formulation (F8) at different concentrations (µg/mL). Data are presented as mean ± SD (*n* = 4). **p < 0.0001 versus the control group (0 µg/mL); NS: not significant.

To confirm the safety of this drug delivery system on human skin cells, the cytotoxicity of the optimized formulation (F8) containing 8% DBA was evaluated using the MTT assay. Different concentrations of F8 (ranging from 50 to 800 µg/mg) were prepared and tested to assess their potential cytotoxicity on human skin cells in monolayer culture. After 48 h of incubation, the results demonstrated that cell viability was not significantly affected up to 100 µg/mg of F8 (equivalent to 8 µg/mg DBA) (Fig. 11). These findings indicate that the F8 formulation containing 8% DBA is safe for human skin cells within this concentration range.

#### Histopathological studies

**Fig. 12:**
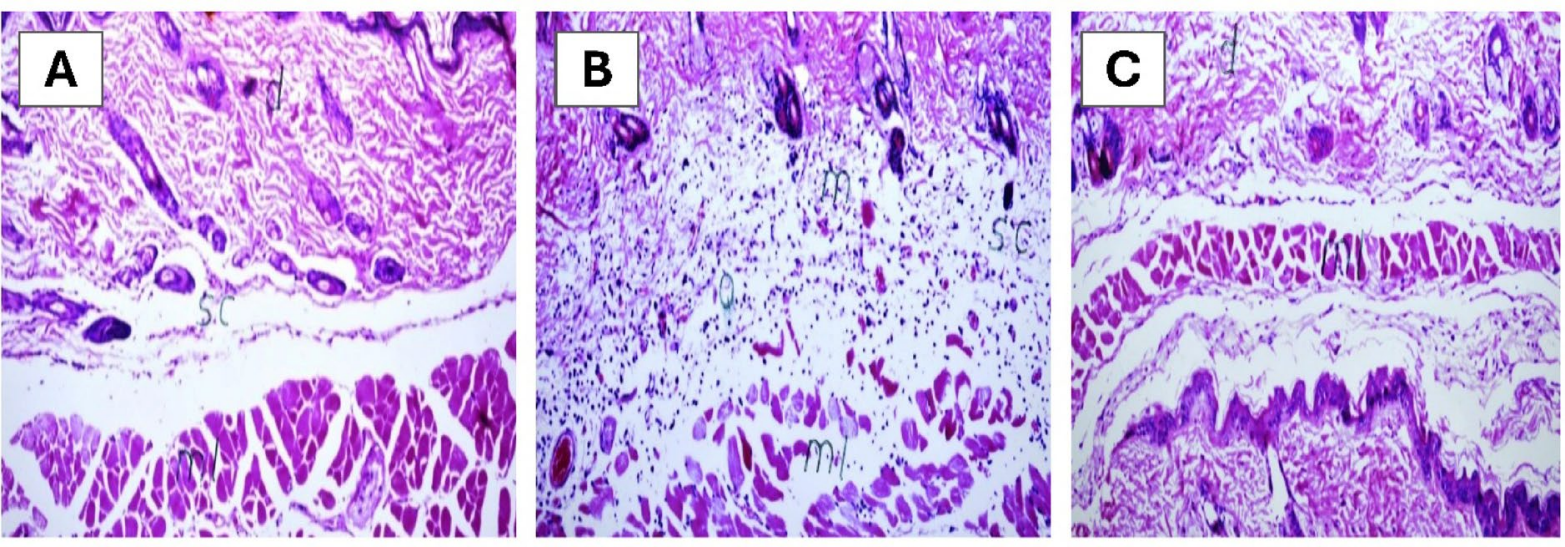
Histological cross-sections of rat skin. (**A**) Normal group, (**B**) standard irritant-treated group, (**C**) optimized DBA oleogel formulation (F8) showing normal skin architecture.

The histopathological photomicrographs of the different experimental groups, including normal skin (control), formalin-treated skin, and F8-treated skin, are presented in Fig. 12A–C. The photomicrograph of the control group (Fig. 12A) revealed normal skin architecture with well-defined epidermal, dermal, subcutaneous, and muscular layers, indicating the absence of any histopathological alterations. In contrast, the skin of rats treated with formalin solution (standard irritant) showed marked pathological changes (Fig. 12B), including focal necrosis in the epidermis, hyalinization in the underlying dermal tissue, and extensive infiltration of inflammatory cells accompanied by edema extending into the subcutaneous tissue and musculature. On the other hand, the skin samples treated with the optimized formulation (F8) exhibited normal histological features comparable to the control group (Fig. 12C). No inflammatory cell infiltration, edema, or structural alterations were observed in the dermal or epidermal layers. These findings indicate that the topical application of F8 did not induce any histopathological changes or visible signs of skin irritation, confirming the dermatological safety of the developed formulation.

## Conclusion

This study involved the development and optimization of DBA loaded oleogel for sunscreen formulations. The results were evaluated using a full factorial (2^3^) design to determine the optimum DBA loaded oleogel formulation (F8). Formula F8 exhibited the highest spreadability, the highest SPF, and the most effective sunscreen protection. The organoleptic properties of formula F8, including its texture, appearance, and overall sensory appeal, were also highly favorable, contributing to its overall acceptability. These findings suggest that formula F8 is the most effective and stable sunscreen formulation, offering optimum photoprotection and enhanced user application.

## Supplementary Information

Below is the link to the electronic supplementary material.

Supplementary Material 1

## Data Availability

All data generated or analysed during this study are included in this published article and its supplementary information files.
