# Supplementary material for: Development and characterization of dibenzalacetone-loaded oleogels as a potential photoprotective agents for sunscreen formulations
Source: Sci Rep. 2026 Jun 9;16:17844. doi: 10.1038/s41598-026-56234-w (PMC13250150; doi:10.1038/s41598-026-56234-w)
Supplement: Supplementary file 1 — Supplementary Material 1 [file 41598_2026_56234_MOESM1_ESM.pdf]

Design Data

Std	Run	Factor 1: Oil type	Factor 2: SD concentration	Factor 3: DBA concentration	Response 1: SPF	Response 2: sunscreen efficacy
1	1	AO	10.00	4.00	7.7	0.35
10	2	AO	10.00	8.00	13.555	0.23
11	3	JO	10.00	8.00	12.5	0.32
12	4	JO	10.00	8.00	12.4	0.3
14	5	AO	15.00	8.00	18.175	0.2
2	6	AO	10.00	4.00	7.9	0.39
13	7	AO	15.00	8.00	19.1	0.24
3	8	JO	10.00	4.00	6.7	0.47
9	9	AO	10.00	8.00	13.72	0.23
8	10	JO	15.00	4.00	11.2	0.4
16	11	JO	15.00	8.00	14.115	0.27
5	12	AO	15.00	4.00	10.5	0.27
6	13	AO	15.00	4.00	10.76	0.281
4	14	JO	10.00	4.00	6.5	0.43
7	15	JO	15.00	4.00	10.9	0.39
15	16	JO	15.00	8.00	14	0.26

ANOVA for selected factorial model

Response 1: SPF

Source	Sum of Squares	df	Mean Square	F-value	p-value	
**Model**	194.90	6	32.48	42.66	< 0.0001	significant
A-Oil Type	10.72	1	10.72	14.07	0.0045	
B-SD Conc.	48.22	1	48.22	63.32	< 0.0001	
C-DBA Conc.	128.85	1	128.85	169.22	< 0.0001	
AB	0.7854	1	0.7854	1.03	0.3363	
AC	6.22	1	6.22	8.17	0.0188	
BC	0.1131	1	0.1131	0.1485	0.7089	
**Residual**	6.85	9	0.7615			
Lack of Fit	6.28	1	6.28	87.88	< 0.0001	significant
Pure Error	0.5718	8	0.0715			
**Cor Total**	201.75	15				

Factor coding is **Coded**.
Sum of squares is **Type III - Partial**

The **Model F-value** of 42.66 implies the model is significant. There is only a 0.01% chance that an F-value this large could occur due to noise.

P-values less than 0.0500 indicate model terms are significant. In this case A, B, C, AC are significant model terms. Values greater than 0.1000 indicate the model terms are not significant. If there are many insignificant model terms (not counting those required to support hierarchy), model reduction may improve your model.

The **Lack of Fit F-value** of 87.88 implies the Lack of Fit is significant. There is only a 0.01% chance that a Lack of Fit F-value this large could occur due to noise. Significant lack of fit is bad -- we want the model to fit.

Fit Statistics

**User Std. Dev.**	0.8726		**R^2**	0.9660
**Std. Dev.**	0.8726		**Adjusted R^2**	0.9434
**Mean**	11.86		**Predicted R^2**	0.8926
**C.V. %**	7.36		**Adeq Precision**	18.7766

The **Predicted R^2** of 0.8926 is in reasonable agreement with the **Adjusted R^2** of 0.9434; i.e. the difference is less than 0.2.

Adeq Precision measures the signal to noise ratio. A ratio greater than 4 is desirable. Your ratio of 18.777 indicates an adequate signal. This model can be used to navigate the design space.

ANOVA for selected factorial model

Response 2: Sunscreen Efficacy

Source	Sum of Squares	df	Mean Square	F-value	p-value	
**Model**	0.0946	6	0.0158	34.20	< 0.0001	significant
A-Oil Type	0.0263	1	0.0263	57.12	< 0.0001	
B-SD Conc.	0.0105	1	0.0105	22.68	0.0010	
C-DBA Conc.	0.0542	1	0.0542	117.54	< 0.0001	
AB	5.062E-06	1	5.062E-06	0.0110	0.9188	
AC	0.0014	1	0.0014	3.01	0.1167	
BC	0.0022	1	0.0022	4.84	0.0553	
**Residual**	0.0041	9	0.0005			
Lack of Fit	0.0014	1	0.0014	4.02	0.0799	not significant
Pure Error	0.0028	8	0.0003			
**Cor Total**	0.0987	15				

Factor coding is **Coded**.
Sum of squares is **Type III - Partial**

The **Model F-value** of 34.20 implies the model is significant. There is only a 0.01% chance that an F-value this large could occur due to noise.

P-values less than 0.0500 indicate model terms are significant. In this case A, B, C are significant model terms. Values greater than 0.1000 indicate the model terms are not significant. If there are many insignificant model terms (not counting those required to support hierarchy), model reduction may improve your model.

The **Lack of Fit F-value** of 4.02 implies there is a 7.99% chance that a Lack of Fit F-value this large could occur due to noise. Lack of fit is bad -- we want the model to fit. This relatively low probability (<10%) is troubling.

Fit Statistics

**User Std. Dev.**	0.0215		**R^2**	0.9580
**Std. Dev.**	0.0215		**Adjusted R^2**	0.9300
**Mean**	0.3144		**Predicted R^2**	0.8672
**C.V. %**	6.83		**Adeq Precision**	17.5087

The **Predicted R^2** of 0.8672 is in reasonable agreement with the **Adjusted R^2** of 0.9300; i.e. the difference is less than 0.2.

Adeq Precision measures the signal to noise ratio. A ratio greater than 4 is desirable. Your ratio of 17.509 indicates an adequate signal. This model can be used to navigate the design space.

Solutions

10 Solutions found

Solutions for 2 combinations of categoric factor levels

Number	Oil Type	SD Conc.	DBA Conc.	SPF	Sunscreen Efficacy	Desirability	
**1**	**AO**	**15.000**	**8.000**	**18.011**	**0.211**	**0.937**	**Selected**
2	AO	15.000	7.976	17.971	0.211	0.934	
3	AO	15.000	7.898	17.838	0.213	0.926	
4	AO	14.737	8.000	17.813	0.212	0.926	
5	AO	15.000	7.849	17.755	0.213	0.921	
6	AO	14.562	8.000	17.682	0.213	0.919	
7	JO	15.000	8.000	14.684	0.274	0.686	
8	JO	15.000	7.955	14.636	0.276	0.682	
9	JO	14.495	8.000	14.395	0.277	0.669	
10	JO	14.435	8.000	14.361	0.277	0.667	
